# Risk factors for infertility and barriers to treatment in Tanzania: a survey and medical records study

**DOI:** 10.4314/ahs.v23i4.50

**Published:** 2023-12

**Authors:** Emily A Groene, Cyrialis Mutabuzi, Dickson Chinunje, Ester Shango, Mkhoi L Mkhoi, Susan M Mason, Shalini Kulasingam, Charles R Majinge

**Affiliations:** 1 Division of Epidemiology and Community Health, School of Public Health, University of Minnesota, 1300 S 2nd Street, Suite 300, Minneapolis, United States; 2 Dodoma Christian Medical Center, Ntyuka, Dodoma, Tanzania; 3 Benjamin Mkapa Hospital, P.O. Box 11088, Inside University of Dodoma (UDOM) area, Dodoma, Tanzania

**Keywords:** Infertility, Infection, Tanzania

## Abstract

**Background:**

The burden of infertility is serious for women in high-fertility countries.

**Objectives:**

We sought to identify demographic, behavioral/environmental, and reproductive risk factors for various infertility factors (i.e., ovarian, tubal, uterine/cervical, male/other) among women seeking infertility treatment in central Tanzania; to determine the association between pelvic inflammatory disease (PID) and tubal factor infertility (TFI); and to identify barriers to infertility treatment by women's home regional zone.

**Methods:**

We conducted a cross-sectional survey of women seeking infertility treatment in Dodoma, Tanzania from January-March 2020. We surveyed 168 participants aged 18-49 years and reviewed their medical records to confirm infertility status and potential risk factors. We estimated prevalence ratios for factors associated with infertility using logistic regression. Treatment barriers were compared by women's regional zone to see if barriers varied geographically.

**Results:**

The median age of participants was 32 years (range: 18-48). Infertility factors did not vary greatly by patient demographics, behavioral/environmental, or reproductive risk factors. Approximately 31.48% of women had PID diagnoses. Those with PID had 1.94 (95% CI: 1.30, 2.90) times the prevalence of TFI diagnosis as those with other infertility factors, after adjusting for age, pesticide use, alcohol use, age at sexual debut, prior obstetric events, and family history of infertility. Logistical barriers to treatment, such as time and cost, were more frequently reported than emotional, stigma, or other barriers, regardless of regional zone.

**Conclusions:**

PID was strongly associated with TFI after adjustment for confounders. Infertility treatment access due to cost remains a challenge in Tanzania.

## Introduction

In the African infertility belt, from Gabon in the west to Tanzania in the east, infertility is highly prevalent 1. Although Tanzania has a total fertility rate above the global average (2.68 children per woman) 2, the prevalence of primary infertility in Tanzania (having no children to date) is 2%, second only to Uganda (3%) in the East African region 3. Secondary infertility (the inability of a woman with a prior pregnancy to have an additional pregnancy) in Tanzania is estimated to be the highest in the East African region at 18% 3,4.

Biological and environmental factors contributing to infertility are mainly identified by studies in wealthier countries with lower fertility rates and later postponement of childbirth 5. Sexually-transmitted infection (STI) leading to pelvic inflammatory disease is hypothesized to be a leading cause of infertility in sub-Saharan Africa 1. Although pelvic inflammatory disease (PID) is an established risk factor for tubal factor infertility (TFI) 6, its association with TFI has not been analysed in the African infertility belt taking into account other risk factors 7. PID's hypothesized effect on TFI may be overestimated because of confounders. The risk factor profile for women experiencing infertility may differ in the African fertility belt due to difference in background STI burden, obstetric complications, smoking and alcohol consumption, obstetric history, contraception use, body-mass index (BMI), age at sexual debut and pregnancy, and lower access to STI screening and medical care 7,8.

In settings where child-bearing is highly valued, infertility may seriously impact how women are treated, resulting in individual psycho-social and socio-economic implications and making infertility treatment particularly important 7. Yet, barriers to infertility treatment have rarely been examined in high-fertility contexts 9. Psychological/emotional barriers, knowledge barriers, logistical barriers, and stigma or cultural barriers have been identified in low-fertility contexts 10, but they have not been quantified in high fertility contexts. Furthermore, there is no analysis of barriers to infertility treatment faced by women by geographic residence, an important consideration given potential logistical challenges for women residing far from treatment. Studies of barriers to healthcare access in sub-Saharan Africa show that geographic and transportation barriers, including inconvenient treatment location, transportation costs, and poor road conditions, may differ by region 11.

This study's objectives were to: (i) identify demographic, behavioral and reproductive risk factors for various infertility factors (ovarian, tubal, uterine/cervical, male/other) among women presenting for infertility treatment in central Tanzania; (ii) determine the association between PID and TFI; and (iii) identify barriers to infertility treatment overall and by women's regional zone of residence.

## Methods

### Study design and setting

We conducted a cross-sectional survey of women of reproductive age (18-49 years) presenting for infertility treatment at the Dodoma Christian Medical Center (DCMC) in Dodoma, Tanzania from January-March 2020. DCMC offers infertility counseling and treatment in the reproductive and child health clinic, established in 2008 and serving over 36,000 patients per year.

### Study participant sampling (inclusion and exclusion criteria)

women aged 18-49 years attending a medical exam while seeking infertility treatment were eligible for the study. Only women who completed their exam were retained in the analysis sample. We excluded women not at risk of pregnancy due to current contraceptive use, sterilization, hysterectomy, or other medical procedures preventing pregnancy. Women who had diminished capacity to consent were excluded. All participants were surveyed by trained medical students while waiting for their appointment, and participants' medical records were reviewed after consultations to confirm medical conditions reported in the survey, other existing health conditions, and infertility factor. Surveys were translated into Kiswahili and pre-tested by four patients and two staff members for comprehension, length, and acceptability. We made minor changes to adapt the demographic questions to the Tanzanian context.

### Diagnostic measures

Clinical infertility was defined by “the failure to achieve a clinical pregnancy after 12 months or more of regular unprotected sexual intercourse,” based on the WHO definition 12. Female infertility was disaggregated into primary infertility (infertility in a woman who has never had a clinical pregnancy) and secondary infertility (infertility in a woman who has had at least one clinical pregnancy) 13. Infertility status was determined by the treating physician in patient consultations.

TFI, the primary infertility factor outcome of interest, was diagnosed through hysterosalpingogram (HSG). Uterine/cervical factor infertility was diagnosed by uterine structural anomalies identified in pelvic ultrasounds. Ovulatory factor infertility was diagnosed by hormone testing, diagnosis of polycystic ovarian syndrome (PCOS), and/or identification of polycystic ovaries on ultrasound. Other forms of infertility were determined by the patient's history, such as infrequent sex or male factor infertility. Whenever the male partner was present for a consultation, male factor infertility was confirmed using semen analysis (sperm analysis).

PID was diagnosed based on any combination of symptoms and signs, including pelvic pain (lower abdominal pain), uterine tenderness (lower abdominal tenderness), tender cervical excitation and tender adnexa of uterus (pelvic pain) that were not explained by an alternative diagnosis, patient history, pelvic ultrasound, and/or a physical exam 14.

### Barriers to treatment

Barriers to treatment were informed by studies of infertility in South Africa, Europe, and Iran 10,15,16. These barriers were categorized as logistical, stigma, emotional, and other barriers. We adapted barriers to the Tanzanian context (Table 1). Survey participants were asked if they had ever experienced barriers when seeking treatment for infertility. If they responded affirmatively, they reported all barriers they experienced and identified the most important barrier.

**Table 1:** Barriers to infertility treatment

Barrier Category	Barrier reported	Source (s)
**Emotional** **Barriers**	Worry about treatment comfort / side effects	Domar 2012, Mosalanejad 2013
	Distrust treatment	Dyer 2002
	Worry treatment might fail	Domar 2012, Dyer 2002, Mosalanejad 2013
**Stigma** **Barriers**	Family stigma	Dyer 2002, Mosalanejad 2013
	Community stigma	Dyer 2002, Mosalanejad 2013
**Logistical** **Barriers**	Treatment cost	Domar 2012, Mosalanejad 2013
	Travel cost	Dyer 2002
	Childcare cost	Pilot testing in Tanzania
	Overcrowded	Pilot testing in Tanzania
	Time	Domar 2012, Dyer 2002, Mosalanejad 2013
	Distance	Dyer 2002, Mosalanejad 2013
	Other logistics	Pilot testing in Tanzania
**Other** **Barriers**	Prefer traditional medicine	Dyer 2002, Mosalanejad 2013
	Patient sick	Pilot testing in Tanzania
	Male factor untested	Mosalanejad 2013, Pilot testing in Tanzania
	Unaware of treatment	Domar 2012, Dyer 2002, Mosalanejad 2013
	Concerns about treatment quality	Dyer 2002, Mosalanejad 2013
	Husband refuses her to seek treatment	Mosalanejad 2013

### Ethical principles

This study was approved by the National Institute for Medical Research in Tanzania (NIMR/HQ/R.8a/Vol. IX/3298) and the University of Minnesota's Institutional Review Board (STUDY00007548). Informed consent was obtained from all participants in the study, and confidentiality was maintained using unique identifiers for patient records and survey results. There were no costs for participation.

### Statistical analysis

Descriptive statistics were presented for associated demographic and behavioral/environmental characteristics, reproductive risk factors and individual barriers to treatment. Risk factors for infertility included in analyses were identified a priori based on a literature review of low and middle-income and high-income countries.

All analyses were carried out using Stata 15.1 (StataCorp.2017, College Station, Texas, USA). Multivariate logistic regression was used to estimate prevalence ratios of PID and TFI by converting prevalence odds ratios to prevalence ratios using the adjrr command in Stata 17. Regression model results were disaggregated by primary and secondary infertility. We controlled for participant age (age 30-34 was the referent) 18; prior exposure to pesticides 19; alcohol use 19; age at sexual debut 20; experience of prior obstetric events, including caesarean sections, hemorrhaging during or after delivery, stillbirth or miscarriage, dilation and curettage 20; and genetic predisposition for infertility defined by having female family members who were also unable to conceive 18.

Barriers were compared by region of origin. We hypothesized that geographic and transportation-related barriers would be greater for women living outside the Central regional zone. Regional zones were defined by the DHS Program's six ecological/geographical zones for Tanzania 21, with the exception of one woman from Lindi region added to the Coastal regional zone.

## Results

Of the 168 women surveyed, 162 women had medical records available and were included in the study. Most participants experienced ovarian factor infertility (n=123, 75.9%) and tubal factor infertility (TFI) (n=57, 35.2%). Many were diagnosed with more than one infertility factor (n=70, 43.21%). Most women were between the ages of 30-39 (median age=32). The study included women from 13 regions, but most resided in the Central regional zone in the Dodoma region (n=132, 79.6%). Participants were highly educated, with 51 (31.5%) women having completed any secondary education and 77 (47.5%) having completed any tertiary education. Most participants were health workers (n=58, 35.8%), followed by other professionals (n=39, 24.1%).

### Demographic characteristics by infertility factor

Demographic characteristics are presented in Table 2 by infertility factor. Women with TFI were most commonly in the 18-29 and 35-39 age ranges (16, 28.1% each), while women with ovarian factor infertility (43, 35%) were most often 18-29 years old, and women with uterine factor (12, 30%) and male or other factors (10, 45.5%) were most often 30-34 years old. The greatest proportion of women with each infertility factor had tertiary education or higher (ranging from 38.6-63.6% for each category). However, a greater share of the 57 women with TFI had primary schooling or less (n=16, 28.1%) compared to the 105 women with other infertility factors (n=18, 17%).

**Table 2:** Demographic characteristics by infertility factor*

Infertility Factor*	Tubal n=57	Ovarian n=123	Uterine n=40	Male / Other n=22
**Age**				
18-29	16 (28.07%)	43 (34.96%)	8 (20.00%)	7 (31.82%)
30-34	15 (26.32%)	30 (24.39%)	12 (30.00%)	10 (45.45%)
35-39	16 (28.07%)	35 (28.46%)	9 (22.50%)	3 (13.64%)
40-49	10 (17.54%)	15 (12.20%)	11 (27.50%)	2 (9.09%)

**Regional Zone**				
Coastal	4 (7.02%)	11 (8.94%)	4 (10.00%)	1 (4.55%)
Northern Highland	2 (3.51%)	4 (3.25%)	2 (5.00%)	-
Lake	5 (8.77%)	8 (6.50%)	1 (2.50%)	-
Central	46 (80.70%)	100 (81.30%)	33 (82.50%)	21 (95.45%)

**Transport to clinic**				
Public	48 (84.21%)	99 (80.49%)	32 (80.00%)	15 (68.18%)
Private	8 (14.04%)	22 (17.89%)	6 (15.00%)	6 (27.27%)
Other	1 (1.75%)	2 (1.63%)	2 (5.00%)	1 (4.55%)

**Education level**				
Primary or less	16 (28.07%)	26 (21.14%)	9 (22.50%)	1 (4.55%)
Secondary	19 (33.33%)	41 (33.33%)	6 (15.00%)	7 (31.82%)
Tertiary	22 (38.60%)	56 (45.53%)	25 (62.50%)	14 (63.64%)

**Marital status**				
Single	7 (12.28%)	17 (13.82%)	5 (12.50%)	3 (13.64%)
Married	50 (87.72%)	106 (86.18%)	34 (85.00%)	18 (81.82%)
Separated/divorced or	-	-	1 (2.50%)	1 (4.55%)
widowed				

**Partner present**	11 (19.30%)	15 (12.20%)	5 (12.50%)	5 (22.73%)

**Occupation**				
Teacher	9 (15.79%)	20 (16.26%)	10 (25.00%)	4 (18.18%)
Health worker	22 (38.60%)	45 (36.59%)	9 (22.50%)	5 (22.73%)
Self-employed	4 (7.02%)	3 (2.44%)	3 (7.50%)	
Student	2 (3.51%)	4 (3.25%)	1 (2.50%)	2 (9.09%)
Unemployed	4 (7.02%)	7 (5.69%)	2 (5.00%)	
First responder/ military	3 (5.26%)	12 (9.76%)	4 (10.00%)	1 (4.55%)
Hospitality		2 (1.63%)	1 (2.50%)	1 (4.55%)
Laborer				1 (4.55%)
Other professional	13 (22.81%)	30 (24.39%)	10 (25.00%)	8 (36.36%)

* Women may experience more than one factor

### Behavioral/environmental characteristics by infertility factor

Self-reported sexual behavior varied between women diagnosed with different infertility factors (Table 3). Women experiencing uterine factor (n=29, 72.5%) and ovarian factor infertility (n=84, 68.29%) were most likely to report frequency of sex as more than once a week, followed by TFI (n=38, 66.7%) and male or other infertility factor (n=13, 59%). The greatest share of women who reported having sex less than once a month had male or other infertility (n=5, 22.7%), and ovarian factor infertility (n=18, 14.6%). Most women in all infertility factor groups reported having 1-2 lifetime sexual partners (range from 45.5-49.1%). Age at sexual debut was most often reported between the ages of 19-24 for women with all infertility factors (range: 45.5%-57.5%).

**Table 3:** Behavioral/environmental and reproductive risk factors by infertility factor*

Factor*		Tubal factor n=57	Ovarian factor n=123	Uterine factor n=40	Male / Other n=22
**Behavioral / Environmental Risk Factors**					

Prior pesticide use		3 (5.26%)	7 (5.69%)	1 (2.50%)	2 (9.09%)

Any alcohol use		10 (17.54%)	30 (24.39%)	9 (22.50%)	5 (22.73%)

	Age at sexual debut				
<15		3 (5.26%)	4 (3.25%)	2 (5.00%)	-
15-18		10 (17.54%)	26 (21.14%)	9 (22.50%)	6 (27.27%)
19-24		31 (54.39%)	65 (53.66%)	23 (57.50%)	10 (45.45%)
≥25		3 (5.26%)	15 (12.20%)	4 (10.00%)	3 (13.64%)
missing		10 (17.54%)	12 (9.76%)	2 (5.00%)	3 (13.64%)

	Lifetime sexual partners				
0		1 (1.75%)	1 (0.81%)		
1-2		28 (49.12%)	60 (48.78%)	19 (47.50%)	10 (45.45%)
3-5		16 (28.07%)	43 (34.96%)	16 (40.00%)	7 (31.82%)
> 5		5 (8.77%)	11 (8.94%)	3 (7.50%)	2 (9.09%)
missing		7 (12.28%)	8 (6.50%)	2 (5.00%)	3 (13.64%)

	Frequency of sex				
More than once a week		38 (66.67%)	84 (68.29%)	29 (72.50%)	13 (59.09%)
Once a week		10 (17.54%)	8 (6.50%)	3 (7.50%)	2 (9.09%)
2-3 times a month		1 (1.75%)	13 (10.57%)	5 (12.50%)	2 (9.09%)
Less than once a month		8 (14.04%)	18 (14.63%)	3 (7.50%)	5 (22.73%)

	BMI category				
Under 25		15 (30.61%)	33 (30.84%)	9 (28.13%)	7 (36.84%)
25-29		19 (38.78%)	35 (32.71%)	11 (34.38%)	9 (47.37%)
30 and over		15 (30.61%)	39 (36.45%)	12 (37.50%)	3 (15.79%)

Has sex in fertile period		44 (77.19%)	94 (76.42%)	36 (90.00%)	15 (68.18%)

**Reproductive/Genetic Health Factors**					

Prior pregnancies					
None		13 (22.81%)	36 (29.27%)	10 (25.00%)	7 (31.82%)
1		22 (38.60%)	32 (26.02%)	12 (30.00%)	6 (27.27%)
2-3		19 (33.33%)	45 (36.59%)	11 (27.50%)	7 (31.82%)
4-9		3 (5.26%)	10 (8.13%)	7 (17.50%)	2 (9.09%)

Had care in last pregnancy		31 (54.39%)	60 (48.78%)	17 (42.50%)	13 (59.09%)

Experienced fetal losses		27 (47.37%)	53 (43.09%)	24 (60.00%)	9 (40.91%)

Prior obstetric event		26 (45.61%)	55 (45.08%)	25 (62.50%)	12 (54.55%)

Prior contraception use		14 (24.56%)	31 (25.20%)	4 (10.00%)	6 (27.27%)

Prior PID		27 (47.37%)	41 (33.61%)	10 (25.64%)	7 (31.82%)

Family member infertile		16 (28.07%)	32 (26.02%)	13 (32.50%)	3 (13.64%)

* Women may experience more than one factor

### Reproductive and genetic characteristics by infertility factor

Women with uterine factor infertility experienced the greatest proportion of fetal losses (n=24, 60%). They were also most likely to have experienced an obstetric event (n=29, 73%). Women with male or other infertility factors made up the greatest share of women with no prior pregnancies (n=7, 31.8%). About 25% of women with each infertility factor had ever used contraception, except women with uterine factor infertility, who only had 10% prior contraception use. Women with TFI had a higher likelihood of prior PID diagnosis (n=27, 47.37%) than women in other groups.

### PID and TFI

The prevalence of PID in all women was 31.48%, which was slightly higher among women with primary infertility (34.94%), compared to secondary infertility (27.85%). Crude and adjusted prevalence ratios (cPR and aPR) of PID and TFI in women with primary and secondary infertility are presented in Table 4. Overall, the relationship between PID and TFI was stronger for women experiencing primary TFI compared to secondary TFI.

**Table 4:** Pelvic Inflammatory Disease by infertility type and Tubal Factor Infertility (TFI)

	All infertility (n=162)	Primary infertility (n=83)	Secondary infertility (n=79)
**PID Prevalence**	31.48%	34.94%	27.85%
**Multivariate Logistic Regression**			
**Unadjusted models**			
		**Crude Prevalence Ratio (95% CI)**	
All TFI		1.94 (1.30-2.90)	
Primary TFI		2.59 (1.44-4.64)	
Secondary TFI		1.44 (0.79-2.61)	
**Adjusted models** *			
		**Adjusted Prevalence Ratio (95% CI)**	
**All** TFI		1.73 (1.10-2.73)	
Primary TFI		2.41 (1.24-4.70)	
Secondary TFI		1.28 (0.65-2.51)	

* Adjusted for participant age, exposure to pesticides, alcohol use, age at sexual debut, experience of obstetric events, and family history of infertility.

### Barriers to treatment

Logistical barriers were most common, including treatment, travel, or childcare cost; time for treatment; distance to the facility; and overcrowding at the facility. Logistical barriers were experienced by 99 women surveyed (61.1%). Only 10 women (6.2%) in the sample reported experiencing emotional barriers, such as concerns about treatment comfort, distrust in the treatment, or worry that the treatment might fail. Five (3%) women reported other barriers, such as concerns about treatment quality, preference for traditional medicine, husband refusing her treatment, other illness, or lack of awareness of treatment. Finally, women were least likely to report stigma barriers from either the family or the community (3, 1.9%). Of the 162 women surveyed, 45 women (27.8%) reported experiencing no barriers to treatment.

### Most important barriers

**Figure 1:**
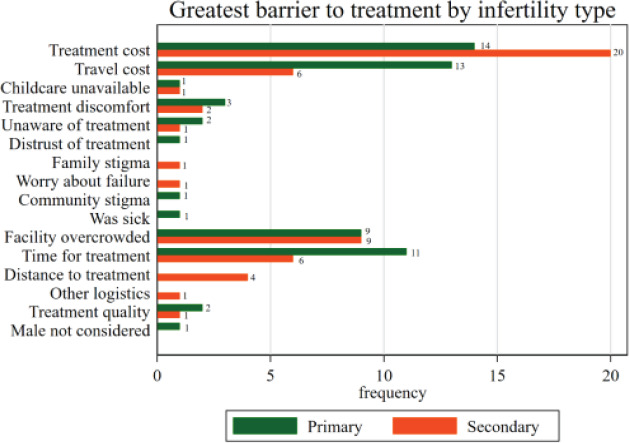
Greatest barrier to treatment by infertility type

Women were also asked which barrier was most important to them. The most important barriers reported did not vary greatly by primary or secondary infertility. Treatment cost was cited as the most important barrier for women participating in the study, both for those with primary (n=14) and secondary (n=20) infertility (Figure 1 – Greatest barrier to treatment by infertility type). Travel cost was the next most important barrier among women with primary infertility (n=13), while facility overcrowding was more important for women with secondary infertility (secondary n=9, primary n=9).

### Differences in barriers by regional zone

Representation by barrier type paralleled the sample's geographic distribution, with the largest group in each barrier category residing in the Central regional zone. Logistical barriers were most often reported by women across regional zones, including 88 women from the Central regional zone (63.3%), 9 women (69.2%) from the Coastal regional zone, and 3 women each from the Northern Highland (60%) and Lake regional zones (30%). Women reporting non-logistical barriers were few, with emotional barriers distributed across Central (5, 50%), Lake (3, 30%), and Coastal (2, 20%) regional zones, stigma barriers reported in Central (2, 66.7%) and Northern Highland (1, 33.3%) regional zones, and other barriers in Lake (1, 9.1%) and Central (4, 2.9%) regional zones.

## Discussion

### Main findings

Our study demonstrates a strong association between PID and TFI after accounting for demographic, behavioral and reproductive characteristics. We found a high prevalence of PID among women in the overall sample (31.5%) and by primary (34.9%) and secondary infertility (27.9%). Barriers to treatment did not greatly differ by women's regional zone of origin. Logistical or access-related barriers were considered most important by women seeking treatment, regardless of primary or secondary infertility type.

This study builds upon work by Dhont and colleagues (2011) who examined the risk factor profile of women experiencing secondary infertility in Rwanda 20. Authors studied the relationship between secondary infertility and socio-demographic characteristics, reproductive tract infections, and obstetric and reproductive history. Our study includes both primary and secondary infertility and adjusts for demographic and reproductive characteristics when examining the relationship between PID and TFI.

### Interpretation

Our findings highlight the importance of addressing PID, which is caused by untreated STIs and other reproductive tract infections (RTIs). These infections are usually treated based on syndromic management (detected by urethral and vaginal discharge symptoms) in Tanzania and many parts of sub-Saharan Africa 22. This approach cannot detect asymptomatic infections and may result in overuse of antibiotics and limited disease surveillance 23. Improved prevention, detection, and treatment of STIs/RTIs may reduce the risk of TFI in similar populations. In more highly resourced settings, testing for STIs is part of standard of care for infertility treatment 24. Incorporating discreet STI testing as standard of care for infertility treatment may reach a population at higher risk of STIs, women engaging in sexual intercourse without barrier contraception to conceive.

Ovarian factor infertility was the most common factor among women in this study, which may be due to older age (median age=32). Most women in the study were highly educated and may have intentionally delayed child-bearing to pursue education or careers. The share of participants with secondary education (31%) and tertiary education (48%) is higher than the general population, where the net secondary enrolment rate for females was 27% in 2018 and where 1.8% of women over the age of 25 had completed any post-secondary education as of 2012 25. Diminished ovarian reserve due to advanced maternal age or early ovarian aging is a challenge in low resource settings with limited access to assisted reproductive technologies 26. Fortunately, ovulatory dysfunction not due to ovarian reserve may be treated by ovulatory induction, which is simple and safe to administer in low resource settings compared to surgical treatment for other infertility factors 27.

Our hypothesis that barriers would differ by regional zones outside of Central Tanzania was not supported by our data, possibly because of our small sample size. Barriers related to treatment access were most often cited by participants, regardless of their residence. We might expect more variability in barriers in a larger sample of women residing outside the Central regional zone. However, women within the Central regional zone also reported logistical barriers to infertility such as travel and treatment cost, highlighting the importance of treatment access. While infertility can seriously impact how women are perceived in high-fertility settings, our analysis suggests that infertility stigma from family and the community are not important barriers 8. It is possible that stigma is common but serves as a motivator for treatment rather than a barrier.

In sub-Saharan Africa, infertility is largely viewed as a woman's issue. Only 24 women (14.8%) were accompanied by a partner on the day of the survey, indicating that few partners were examined for the possibility of male factor infertility. While both men and women contribute to infertility in sub-Saharan Africa, women tend to endure the blame 28. In a study in Nigeria, male factor infertility accounted for 42% of couples attending an infertility clinic, and 21% of couples experienced a combination of male and female factors. This suggests that partners of participants in our study who were diagnosed with a female infertility factor could also be experiencing undiagnosed male factor infertility. Thus, increased focus on testing and treatment for male infertility could improve not only the social impact on women, but also infertility treatment outcomes for couples.

## Strengths and limitations

This study has several strengths, including its focus on an understudied, clinically important problem in Tanzania. First, few studies of infertility risk factors have been conducted in sub-Saharan Africa 1. Further, this is one of the first studies of its kind to assess potential barriers to infertility treatment in Tanzania, a practical consideration. Secondly, we combined thorough chart review with survey administration to validate participant responses regarding health history. Finally, despite resource constraints, this study relies upon high standards for diagnosis of each infertility factor, including the use of ultrasound, hysterosalpingography and laboratory testing of hormone levels, which has not been made explicit in other studies of infertility in sub-Saharan Africa. However, additional testing that was unavailable at the study site would have improved accuracy further, such as laparoscopy for confirmation of tubal factor infertility. Testing for bacterial infections leading to PID was not conducted for all women participating in the study, and infection types were not included among risk factors, despite evidence for the association between prior chlamydia and mycoplasma infections with tubal factor infertility in low-resource settings 29.

This study has several limitations. Women attending infertility treatment may not be generalizable to the population of all women experiencing infertility, a common challenge for infertility studies 30. Barriers identified among women who receive infertility treatment may differ from barriers experienced by those without treatment. To address potential recall bias in the survey, we validated responses using existing medical records. Our approach may miss risk factor diagnoses made at other health facilities, so ours is a conservative measure of the association of these factors with infertility if diagnoses are missing evenly across the population. Participant recall could also be biased due to social desirability regarding sexual behavior, resulting in underreporting some risk factors. We recorded instances when women declined to respond, including age at sexual debut (9.3% missing), number of lifetime sexual partners (6.2% missing), sex during the fertile period (1.9% missing), and prior fetal losses (27.2% missing). Among clinical variables, only BMI was not consistently recorded in the medical record (14.2% missing).

## Conclusion

In conclusion, our study highlights a strong association between PID and TFI. However, TFI due to infection was not the sole driver of infertility in this setting; a substantial number of women were affected by ovarian factor and other infertility factors. We identified important logistical treatment barriers among women seeking infertility treatment, highlighting the importance of expanded treatment access. More population-based studies of infertility in low-resource contexts may better discern patterns in risk factors and infertility factor types.
